# Role of Extra-anatomic Ascending to Descending Bypass in Complex Thoracic Aortic Pathology

**DOI:** 10.1055/s-0041-1739483

**Published:** 2021-12-28

**Authors:** Mariano Camporrotondo, Sebastian Pagni

**Affiliations:** 1 Department of Cardiac Surgery, Instituto Cardiovascular Buenos Aires, Buenos Aires, Argentina; 2 Department of Cardiac Surgery, Baptist Health Louisville, Louisville, Kentucky

**Keywords:** TEVAR infection, complex aortic coarctation, ascending–descending bypass

## Abstract

Complex pathology of the distal arch and proximal descending thoracic aorta is usually approached by stent endografting or in situ graft replacement. Oftentimes, these options are not feasible due to unfavorable anatomy, multiple previous procedures, active infection, or presence of concomitant cardiac disease. Thoracic aortic extra-anatomic bypass, as part of an open surgical strategy, is a useful and often the only curative option left for the treatment in these patients. Herein, we describe two cases that illustrate the utility of extra-anatomic thoracic aortic bypass for complex aortic disease.

## Introduction

Currently, both open in situ grafting and thoracic endovascular aortic repair (TEVAR) procedures are the first choice to treat complex aortic pathology such as aortopulmonary/esophageal fistulas, recurrent coarctation, aneurysms, and some infected vascular grafts. But in many cases, a more complex single or staged open procedure using thoracic aortic bypass is the only curative option left, representing a decision-making dilemma. We describe two clinical cases of thoracic ascending to descending aortic bypass (single and staged) used to treat unusual recurrent aortic problems that illustrate the usefulness of this strategy in complex aortic pathology.

## Case Presentation

### Case 1

**Fig. 1:**
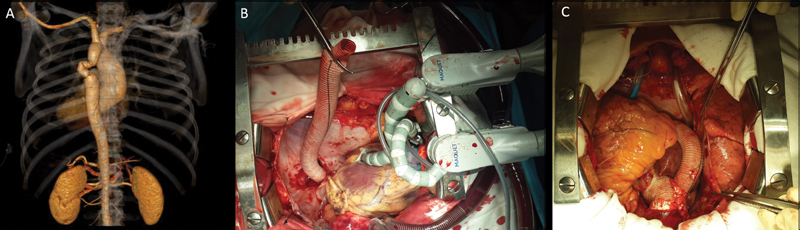
( **A** ) Posterior view of preoperative computed tomography scan that shows severe kinking of the descending aorta Dacron graft. ( **B** ) Surgical view of the descending aorta anastomosis with the heart stabilizers in place, and ( **C** ) after the surgery is completed with the ascending to descending bypass graft.

A 46-year-old male presented with severe intermittent claudication and dyspnea. His medical history included severe aortic valve stenosis, bicuspid aortopathy, and uncontrolled hypertension. He had aortic coarctation repair at the age of 11 years with a 16-mm Dacron graft. Computed tomography (CT) scan revealed a 5-cm ascending aortic aneurysm and a severely kinked Dacron graft in the descending aorta ( Fig. 1A ). A one-stage open procedure was indicated to fix both ascending and descending aortic problems given endografting was unsuitable. A median sternotomy was performed and cardiopulmonary bypass was established. Beating heart techniques allowed us to expose the descending thoracic aorta through the posterior pericardium by retracting the heart cephalad with a heart positioner. The aorta was controlled with a partially occluding vascular clamp, and an end-to-side graft anastomosis was completed ( Fig. 1B ). The graft was routed posterior to the inferior vena cava and lateral to the right atrium. Then a supracoronary aortic graft (26 mm) and a mechanical aortic valve were implanted. The descending graft was then anastomosed to the lateral aspect of the proximal graft ( Fig. 1C ). Recovery was uneventful with discharge home in 4 days and resolved claudication.

### Case 2

**Fig. 2:**
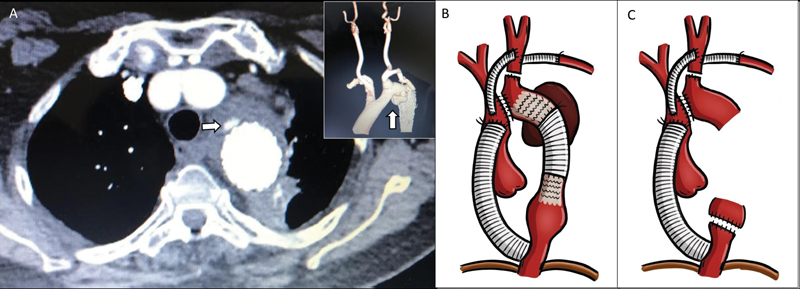
( **A** ) Computed tomography angiography shows mediastinal infection with fat stranding and perianeurysm contrast extravasation (arrow). The three-dimensional reconstruction reveals the proximal false aneurysm (inset—white arrow). ( **B** ) Stage-1 repair with ascending to descending bypass graft, ascending aorta to carotid bypass, previous carotid-subclavian bypass, and arch interruption and resection of the previous stent graft. ( **C** ) Final anatomy after surgical repair with extra-anatomic aortic bypass, stent graft, and descending aorta resection.

A 69-year-old hypertensive female presented with recurrent hemoptysis and pneumonias. She had a history of isthmus coarctation as a child repaired with a patch aortoplasty followed by aneurysm formation repaired with a Dacron tube graft at 46 years of age. She developed severe hemoptysis at 57 years of age, requiring a left subclavian-carotid bypass and stent endografting and fungal suppression therapy for a positive cryptococcus bronchial culture. She was admitted to our service 12 years later with hemoptysis and malaise. The chest CT angiography ( Fig. 2A ) revealed mediastinal contrast extravasation, false aneurysm, and lung parenchyma involvement confirmed later with a bronchoscopy demonstrating blood in the left lower lung bronchi. A two-stage open procedure was performed. Through a median sternotomy, she underwent ascending aorta to left carotid bypass with a 10-mm Dacron graft, ascending-to-descending aorta bypass with a 16-mm Dacron graft using cardiopulmonary bypass and then aortic arch transection ( Fig. 2B ). A week later, she underwent third time left thoracotomy with mediastinal debridement and removal of the aorta and stent-grafts above the new Dacron graft ( Fig. 2C ). Aortic stump coverage with a diaphragmatic flap and segmental lung resection were required. Cultures were negative but she was placed on oral fluconazole suppression therapy. Postoperatively, she developed a chylothorax treated conservatively and was discharged home 21 days after initial surgery. At 2-year follow-up, she was without signs of recurrent infection.

## Discussion

Ascending-to-descending aortic bypass was described in 1980 by Vijayanagar et al 1 in a 30-year-old man with aortic coarctation and severe aortic regurgitation. The repair was done through a median sternotomy from the ascending to the retrocardiac descending thoracic aorta, using a posterior pericardial approach as we described. For decades, the extra-anatomic ascending–descending aortic bypass has been useful to treat various forms of arch/descending aortic disease, mainly recurrent coarctations. 2 3 4 But the advent of thoracic stent endografting (TEVAR) in the early 1990's, and the subsequent facilitation by the evolving technology, made this the treatment of choice for most pathology in the area, including many recurrent coarctations. Moreover, it is also the life-saving first-line treatment for patients with infected grafts and erosion with fistulization and acute bleeding. In situ graft replacement is performed mainly for aneurysmal disease not amenable to endografting, some recurrent coarctations associated with aneurysm or mycotic aneurysms, and graft infection that requires antibiotic soaked grafts or homografts. 5

Lately, the extra-anatomic bypass (EAB) has been reserved for the more complex aortic problems where stenting is not suitable, or there is concomitant proximal aorta or cardiac pathology and/or infected grafts/endografts. The later patients often have graft and/or mediastinal infection and despite the initial palliation and chronic antimicrobial suppression, some patients will require further treatment unless too elderly or sick. 6

A single stage operation (first case) is favored in patients with recurrent coarctations when TEVAR is anatomically unsuitable due to concomitant pathologies of the descending thoracic aorta/arch or associated proximal aortic and cardiac disease that requires repair through a median sternotomy. The EAB avoids an otherwise difficult and high-risk thoracotomy in a second stage and a potentially suboptimal result. A two-stage open surgical repair (second case) is less commonly used but often the only option for curative treatment in cases of graft infection with aortoesophageal, pulmonary-parenchymal or airway fistulization, or mediastinal involvement with false aneurysm. 5 Many patients who are elderly and sick to have this high-risk procedure may opt for palliative care. This approach allows for a sterile redirecting of aortic flow followed by the safer removal of the infected graft and mediastinal debridement in the hostile chest, decreasing the risk of severe bleeding.

In conclusion, these two cases demonstrate the successful surgical management of a spectrum of complex thoracic aortic disease using thoracic aortic EAB. The use of a single versus a two-stage repair should be tailored to the patient's particular aortic process and surgeon's experience.
